# Clinical features of adult patients with allergic parotitis

**DOI:** 10.1016/j.waojou.2023.100864

**Published:** 2024-01-05

**Authors:** Shiyu Gao, Sheng Li, Heming Wu, Yi Yuan, Xu Ding, Jing Zhao, Ailing Wang, Xiumeng Cao, Hongming Du

**Affiliations:** a Department of Oral and Maxillofacial Surgery, Affiliated Stomatological Hospital of Nanjing Medical University, Nanjing, Jiangsu Province, 210029, PR China; b Jiangsu Key Laboratory of Oral Disease, Nanjing Medical University, Nanjing, Jiangsu Province, 210029, PR China; c Jiangsu Province Engineering Research Center of Stomatological Translational Medicine, School of Stomatology, Nanjing Medical University, Jiangsu Province, 210029, PR China

**Keywords:** Adult, Allergic parotitis, Disease attributes, IgE, Parotid disease

## Abstract

**Background:**

Allergic parotitis (AP), due to its non-specific symptoms, frequently poses a diagnostic challenge, leading to cases being overlooked or misdiagnosed by clinicians.

**Objective:**

This study aimed to elucidate detailed clinical characteristics and common diagnostic indicators of AP.

**Methods:**

A comprehensive review and analysis of medical records was conducted from patients diagnosed with AP, encompassing demographic, clinical, and laboratory data, at the Affiliated Stomatological Hospital of Nanjing Medical University between January 2019 and March 2022.

**Results:**

The study enrolled 17 patients, evidenced by an average age of 36.00 ± 12.95 years. Common presentations of AP among the patients included notable symptoms such as parotid gland swelling, associated pain, and xerostomia. Ten patients had other atopic diseases. Palpation revealed the affected parotid glands to be soft and nodular, with an elevated local skin temperature. The unstimulated whole saliva flow rate was decreased. Ultrasonography demonstrated increased volume, reduced echo heterogeneity, and lymph node enlargement in the affected parotid glands. All cases observed increased serum salivary amylase and total IgE levels. Investigation of food allergens and inhaled allergen-specific IgE showed that all patients had suspected food allergies. Food provocation tests (FPT) induced AP in 13 cases, confirming the role of food allergens.

**Conclusion:**

Food allergens are involved in the etiology of AP, underscoring the importance of comprehensive clinical evaluation, including symptoms, signs, and confirmatory auxiliary tests, such as FPT, for accurate diagnosis and differentiation from other salivary gland pathologies.

## Background

Parotid gland swelling arises from diverse etiologies, including acute and chronic infections,^1^ rheumatic diseases,^2^^,^^3^ and radiation damage.^4^ In 1925, Burton-Fanning first identified allergies as a cause of acute parotid gland swelling,^5^ a condition now known as allergic parotitis (AP), further detailed in subsequent case reports.^6^, ^7^, ^8^, ^9^

AP primarily manifests as parotid swelling following allergen exposure, often perplexing patients unaware of their allergen contact.^9^ Despite its rarity, the under-recognition of AP complicates diagnosis, leading allergy physicians to overlook it and oral and maxillofacial surgeons to misconstrue it as chronic obstructive parotitis (COP) or chronic recurrent parotitis (CRP). Highlighting AP's clinical features is crucial to preventing diagnostic errors, underscoring the need for increased awareness among clinicians to consider AP in unexplained acute parotid swelling cases.

## Methods

### Study design and participants

This study was a single-center, retrospective clinical study investigating the characteristics and potential triggers of acute parotid swelling and pain. We reviewed cases from January 2019 to March 2022, identified from the medical records at the Affiliated Stomatological Hospital of Nanjing Medical University. The inclusion criteria for the participants were: adults (18 and older) exhibiting acute parotid swelling, with serum IgE levels exceeding 100 IU/mL and serum salivary amylase over 220 U/L, and a positive response to anti-allergic medication, evidenced by symptom relief within 2 h of administration. Patients were excluded if they were diagnosed with: Sjogren's syndrome (SS), IgG4-related disease (IgG4-RD), COP or CRP, acute infectious parotitis, conditions causing elevated salivary amylase (eg, renal or pancreatic disorders), or those who had undergone specific radiation therapies (including 131I, or radiotherapy targeting the head, neck, or chest). All participants provided informed consent for this retrospective review. This study was approved by the Ethics Committee of the Affiliated Stomatological Hospital of Nanjing Medical University.

### Procedure

Two oral and maxillofacial surgeons reviewed all patient data extracted from medical records. Detailed information on systemic diseases, atopic conditions, and salivary gland discomfort was extracted from the patients' medical histories. Physical examinations had been conducted on the patients' bilateral parotid glands, assessing for swelling, nodularity, texture, and skin temperature. In cases where it was feasible, the affected parotid glands had been manually compressed to evaluate saliva discharge. The unstimulated whole saliva flow rate (UWS) had been measured. Laboratory data were compiled, encompassing results from bilateral parotid gland ultrasounds, complete blood cell counts, total serum IgE levels, serum salivary amylase levels, and allergen-specific IgE screenings for food and inhalants. Anti-allergic treatment had been administered to all patients, with its effectiveness assessed based on symptom relief after 48 h.

Patients with sIgE levels exceeding 100IU/mL or suspected food allergens, in the absence of sIgE results, require skin puncture tests (SPT) for confirmation. SPT outcomes were evaluated according to the ALK-ABELLO criteria for allergen reactivity. Skin reaction was expressed as mean wheal diameter, equal to (maximum transverse diameter D + maximum transverse diameter midpoint vertical diameter)/2. Skin index (SI) = saline diameter ÷ histamine diameter. “-" means SI = 0; "+" means SI < 0.5; "+ +" means 0.5≤SI < 1.0; "+ + +" means 1.0 ≤ SI < 2.0, " + + + +” means SI ≥ 2.0. Foods eliciting strong (++ or +++) reactions in SPT, as well as those yielding negative but questionable results, were subjected to an open food provocation test (FPT) to identify potential allergens triggering AP.^10^ Clinicians and patients were informed about the food types used during the FPT. Patients were instructed to abstain from suspected allergenic foods and anti-allergy medications for 2 weeks before the FPT. The FPT involved oral administration of test samples, with food protein concentrations ranging from 3 to 3000 mg, at 20-min intervals, followed by close observation for any clinical manifestations. The emergence of allergic symptoms, including itchy lips, skin reactions, or throat discomfort within 2 h post-ingestion, was classified as a positive reaction. Apparent swelling and discomfort in the parotid gland prompted blood collection for quantitative serum salivary amylase and IgE assays.

### Statistical analysis

All continuous measurements were presented as mean ± standard deviation (SD). Measurements outside the normal range for laboratory results were also assessed. SPSS (version 22.0) was used to analyze all data.

## Results

This study encompassed 17 patients, 7 males, and 10 females, with a mean age of 36.00 ± 12.95 years (Table 1, Table 2). All patients experienced parotid gland swelling, with no exacerbation of symptoms during meals. Slight pain and xerostomia were reported by 12 patients (71%), as detailed in Table 1, Table 2 Table 1 illustrates that every patient experienced unilateral parotid gland discomfort. Medical histories indicated systemic diseases in 8 patients (Table 2). Of the patients, 10 had histories of atopic diseases: 6 with allergic rhinitis, 4 with eczema, and 1 with asthma. Twelve patients (71%) reported unexplained parotid gland discomfort within the year before seeking medical attention (Table 1, Table 2). None of the patients had experienced recurrent parotid discomfort for periods exceeding 1 year.

**Table 1:**
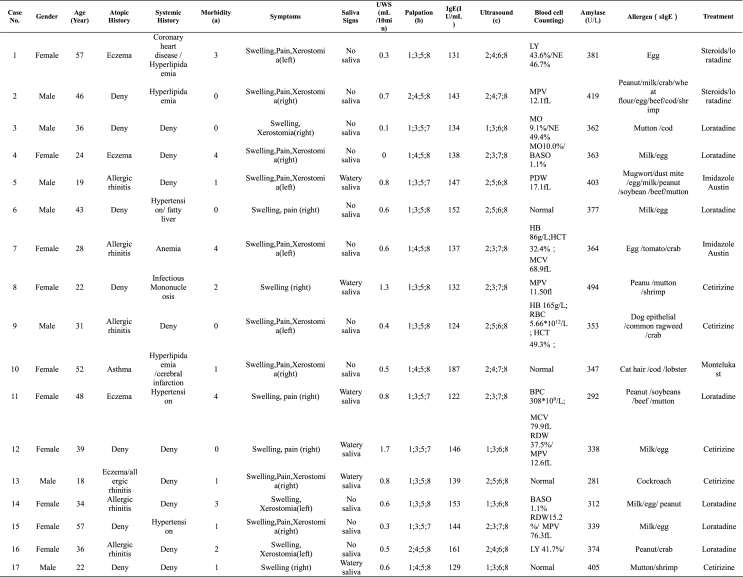
Basics, clinical, laboratory and treatment features of patients.

^a^The times of parotid gland discomfort (1 year prior to onset).

^b^1. no swell; 2. Swell; 3. no nodule; 4. nodule; 5. palpated soft; 6. palpated abnormity; skin temperature (7. normal; 8. slightly high).

^c^Volume (1. normal; 2. enlargement), internal echo (3. normal; 4. low; 5. heterogeneity), lymph nodes (6. normal; 7. enlargement), ducts (8. normal).

**Table 2:** Clinical features of the adults with AP

Item	
Gender	Female:Male = 10:7
Age (Years)	36.0 ± 12.94
Atopic History (n/%)	10/59%
Systemic History (n/%)	8/47%
Morbidity (n/%)	12/71%
Gland involvement	Left:Right = 6:11
**Symptom or Signs (n/%)**	
Swelling	17/100%
Pain	12/71%
Xerostomia	12/71%
No Saliva on the affected side	11/64%
**Palpation (n/%)**	
swell	2/12%
nodule	6/35%
character soft	17/100%
high skin temperature	12/71%

All patients presented with parotid glands that were soft upon palpation. Two cases (12%) were found to have parotid gland swelling, 6 cases (35%) had palpable nodules, and 12 cases (71%) had elevated local skin temperature. In 6 (35%) of the cases, squeezing the affected parotid glands resulted in a minor watery saliva discharge, while 11 cases (65%) showed no saliva (Table 1, Table 2). Regarding the unstimulated whole saliva flow rate, any case with a <1 mL/10 min flow rate was classified as xerostomic.^11^ Of the total cases, 2 exhibited no significant reduction in salivary secretion. In contrast, the other 15 recorded an average flow rate of 0.62 ± 0.41 mL/10 min, falling below the 1 mL/10 min threshold.

As shown in Table 3, ultrasonography of the bilateral parotid glands showed abnormalities in 13 cases (76%), while the remaining 4 cases (24%) were normal. There was volume enlargement of the affected parotid gland in 13 cases (76%). Internal echo was low in 4 cases (24%), with heterogeneity in 4 cases (24%). Lymph node enlargement in the parotid gland was observed in 7 cases (41%). There was no apparent Stensen duct stenosis or dilation in any of the cases. Complete blood cell counts showed abnormalities in 13 cases (76%) (Table 1). The abnormal items detailed in Table 1 were lymphocyte percentage (LY), neutrophil percentage (NE), mean platelet volume (MPV), monocyte percentage (MO), basophilic granulocyte percentage (BASO), platelet distribution width (PDW), hemoglobin (Hb), hematocrit (HCT), mean corpuscular volume (MCV), red blood cell (RBC), blood platelet count (BPC) and red blood cell distribution width (RDW). However, the results of complete blood cell counts did not show some regular pattern. Serum salivary amylase levels were abnormally elevated in all cases, exceeding the normal range (<220 U/L), with an average measurement of 364.94 ± 50.10 U/L. Total serum IgE levels were also found to be abnormally high in all cases, reaching a mean of 142.29 ± 15.57 IU/mL, well above the normal range (<100 IU/mL). Screening results for food allergens and inhaled allergen-specific IgE demonstrated that all patients had suspected food allergies, and 3 (18%) patients had suspected sensitivities to inhaled allergens.

**Table 3:** Ultrasound characters

Ultrasound Character	n/%
Abnormal	13/76%
Volume enlargment	13/76%
Internal echo	
low	4/24%
heterogeneity	4/24%
Lymph nodes enlargement	7/41%

Following anti-allergic interventions, all patients experienced symptom relief. Treatment modalities included steroids (administered to 2 patients), antihistamines (administered to fifteen patients), and leukotriene receptor antagonists (administered to 1 patient), as detailed in Table 1. SPT failed to induce AP symptoms, including parotid gland discomfort. In contrast, FPT triggered AP in 13 (76%) patients, leading to a decrease in total salivary secretion, along with elevated levels of serum salivary amylase and serum IgE (Table 4).

**Table 4:** The results of SPT and FPT.

Case No.	SPT	FPT	Allergen causing AP in FPT	Parotid Symptoms	Saliva Signs	Flow Rates a	Palpation b	IgE^c^	Amalase d
1	Egg/shrimp	Egg	Egg	Swelling, Pain, Xerostomia (left)	No saliva	0.8	1; 3;5; 8	165	312
2	Peanut/milk/crab/wheat flour/egg/beef/cod/shrimp	Milk/crab/wheat flour/egg/beef/shrimp	Milk	Swelling, Pain, Xerostomia (right)	No saliva	0.3	2; 4;5; 8	170	382
			Crab	Swelling, Xerostomia (right)	No saliva	0.5	2; 4;5; 8	132	297
3	Mutton/cod/shrimp	Mutton/cod	Mutton	Swelling, Pain, Xerostomia (right)	No saliva	0.4	1; 3;5; 7	192	378
4	Milk/egg/peanut	–	–	–	Watery saliva	1.7	2; 4;5; 8	–	–
5	Mugwort/dust mite/egg/milk/peanut/soybean/beef/mutton	Egg/peanut/soybean	Soybean	Swelling, Pain, Xerostomia (left)	No saliva	0.7	2; 3;5; 7	194	429
6	Milk/egg	Milk/egg	Egg	Swelling, Pain (right)	No saliva	0.4	1; 3;5; 8	158	352
7	Egg/tomato/crab	Egg/crab	Crab	Swelling, Pain, Xerostomia (left)	No saliva	0.9	2; 4;5; 8	189	268
8	Peanut/mutton/shrimp	Peanut/mutton/shrimp	–	–	Watery saliva	1.3	1; 3;5; 7	–	–
9	Dog epithelial/common ragweed/crab	Crab	Crab	Swelling, Pain, Xerostomia (left)	No saliva	0.7	1; 3;5; 8	193	318
10	Cat hair/cod/lobster	Cod/lobster	Cod	Swelling, Pain, Xerostomia (right)	No saliva	0.4	2; 3;5; 8	223	357
			lobster	Swelling, Xerostomia (right)	No saliva	0.6	1; 3;5; 8	184	285
11	Peanut/soybeans/beef/mutton	Beef/mutton	Beef	Swelling, Pain (right)	Watery saliva	0.7	1; 3;5; 7	171	263
			Mutton	Swelling, Pain, Xerostomia (right)	No saliva	0.5	1; 3;5; 8	187	323
12	Milk/egg/shrimp	Milk/egg	Milk	Swelling, Pain, Xerostomia (right)	No saliva	0.9	1; 3;5; 8	145	298
13	Cockroach	–	–	–	Watery saliva	1.8	1; 3;5; 7	–	–
14	Milk/mutton/egg/peanut	Milk/egg/peanut	Egg	Swelling, Xerostomia (left)	No saliva	0.8	1; 3;5; 8	153	274
			Peanut	Swelling, Pain, Xerostomia (right)	No saliva	0.5	1; 3;5; 8	169	266
15	Milk/egg	Egg	Egg	Swelling, Pain, Xerostomia (right)	No saliva	0.6	1; 3;5; 8	121	285
16	Peanut/crab	Peanut/crab	Peanut	Swelling, Xerostomia (left)	No saliva	0.8	2; 4;5; 8	135	298
			Crab	Swelling, Pain, Xerostomia (right)	No saliva	0.4	2; 4;5; 8	181	332
17	Mutton/shrimp	Mutton/shrimp	–	–	Watery saliva	1.4	1; 3;5; 7	–	–

a Unstimulated whole saliva flow rate（mL/10min）(<1mL/10min).

b Palpation: Swell (1. no swell; 2. swell), nodule (3. no nodule; 4. nodule), character (5. soft; 6. abnormity), skin temperature (7. normal; 8. slightly high).

c Serum total IgE (IU/mL)（the normal range <100IU/mL).

d Serum salivary amylase (the normal range <220 U/L).

## Discussion

Previous studies, including those by Bookman et al have characterized the clinical manifestations of AP, noting its occurrence in individuals ranging from 2.5 to 70 years of age.^6^, ^7^, ^8^, ^9^ A significant proportion of these patients also present with concurrent atopic conditions. AP's presentation can be unilateral or bilateral, with a rapid onset spanning several hours to days. While local pain might be absent, patients often report cheek swelling. Typically, physical examinations indicate soft, non-tender parotid glands. The prompt alleviation of symptoms following anti-allergic interventions serves as a diagnostic indicator. The present study's findings align with existing literature, further enriching AP's clinical and laboratory understanding. Additionally, this research underscores the necessity of distinguishing AP from other conditions with similar presentations during the differential diagnosis process in clinical practice.

In this retrospective analysis, ultrasonography findings indicated mild parotid gland swelling in most AP cases (13/17) included in the ultrasound examination, and lymphadenopathy was documented in 7 cases. These data underscore the potential diagnostic relevance of ultrasonography in AP. Interestingly, as retrieved from records, blood cell counts in AP patients did not exhibit the typical hallmarks of allergic diseases.^12^^,^^13^ This pattern suggests a distinct hematological response in AP, divergent from other allergic conditions, and indicates the limited diagnostic utility of blood cell counts for AP. Medical records revealed a high prevalence of allergic diseases among participants (10/17), aligning with previous literature^9^ and emphasizing the importance of a comprehensive review of patients' past medical histories in clinical assessments.

The parotid gland, known for its serous acini, is the primary producer of salivary amylase.^14^ Clinical data highlighted a consistent observation: individuals documented with AP showed elevated serum salivary amylase levels. This finding serves as objective evidence, suggesting that inflammatory responses, likely due to allergic reactions, may have compromised the integrity of serous acinar cells in the parotid gland, leading to the systemic release of salivary amylase.

AP and mumps both initially present with similar symptoms, including parotid gland swelling, pain, and elevated local skin temperature, making differential diagnosis challenging.^15^ Therefore, a detailed patient medical history, particularly regarding vaccination records and previous mumps episodes, is crucial for accurate diagnosis.^16^ Unlike mumps, AP may manifest as recurrent discomfort in the parotid gland. Mumps, a viral infection, typically induces specific hematological changes such as a decreased white blood cell count and an increased monocyte count, identifiable through blood tests, and its presence can be confirmed via viral nucleic acid detection.^17^ These hematological markers are generally not observed in AP. While both conditions can lead to elevated levels of serum salivary amylase, this parameter alone is insufficient to distinguish between the two. Instead, clinicians should consider the unique blood cell count patterns, such as decreased white blood cell count and increased monocyte count, as well as viral nucleic acid presence in mumps, which are not characteristic of AP. These distinctions are vital for accurate diagnosis and appropriate management.

AP is frequently misdiagnosed as CRP or COP due to overlapping clinical presentations, including recurrent parotid gland swelling and pain. While CRP affects both adults and children, COP predominantly occurs in middle-aged individuals.^18^ The main symptoms in CRP and COP are obstructive symptoms such as repeated swelling and pain in the parotid glands.^19^ Notably, COP is characterized by the secretion of "snow-like" or mucoid saliva upon gland compression, often accompanied by mucus plugs.^20^ Conversely, CRP's radiographic findings reveal punctate and spherical dilations of peripheral ducts with delayed emptying, without significant anomalies in the main and intraglandular ducts.^21^ COP's sialography typically demonstrates localized stenosis or ductal dilation, presenting a "sausage-like" appearance.^22^ In AP, physical examination often reveals increased skin temperature over the parotid area, potentially leading clinicians to misinterpret AP as an acute exacerbation of COP or CRP. This diagnostic modality should be employed judiciously because acute infections contraindicate parotid sialography. Differentiation of AP may be more accurately achieved by assessing serum total IgE and salivary amylase levels, complemented by the patient's response to anti-allergic medications.

The diagnostic landscape for AP is fraught with controversies, particularly regarding its distinction from Eosinophilic Sialodochitis (ES). Both conditions manifest with recurrent salivary gland swelling, pain, xerostomia, a history of allergic diseases, and elevated serum IgE levels, responding favorably to anti-allergic therapies.^23^^,^^24^ However, ES is characterized by a protracted clinical course, local skin itching, the expulsion of string-like mucus rich in eosinophils from salivary ducts, peripheral eosinophilia, and radiographic evidence of ductal dilation.^23^^,^^24^ Given these overlaps with COP,^20^ a potential allergic etiology linking ES and COP warrants further exploration.

Moreover, the clinical presentation of AP in this study, including xerostomia, parotid swelling, pain, and recurrent discomfort, mirrors symptoms observed in SS, IgG4-RD, and radioactive parotitis.^25^, ^26^, ^27^ Consequently, clinicians must exercise discernment when evaluating middle-aged and elderly patients presenting with acute parotid swelling. Comprehensive diagnostic approaches should encompass assessments of serum total IgE, SS-specific antibodies (anti-SSA, anti-SSB, rheumatoid factor), serum IgG4 levels, and a thorough review of any prior radiotherapy to differentiate AP from these mimicking conditions effectively.

Oral allergy syndrome (OAS) is a type I hypersensitivity reaction mainly caused by food allergens, leading to oral mucosal allergic diseases and often accompanied by other allergic diseases such as eczema, asthma, and allergic rhinitis.^28^ Patients with OAS primarily experience edema, oral mucosal congestion, a burning or tingling sensation, tongue and soft palate swelling, throat itching, and pallor of the mucous membranes.^28^ However, standard OAS descriptions in existing literature do not encompass distinct AP symptoms such as parotid gland swelling, pain, and discomfort. This omission likely contributes to the oversight of AP diagnoses among allergy clinicians. Furthermore, with epidemiological data indicating an increase in food allergy prevalence, there may be a corresponding rise in AP incidents. Consequently, we advocate for formally recognizing AP within the OAS categorization to facilitate comprehensive diagnostic and therapeutic strategies.

## Conclusions

Food allergens are involved in the etiology of allergic parotitis. Clinicians diagnosing AP must carefully consider a constellation of clinical symptoms, physical signs, and corroborative diagnostic tests. The FPT stands as a pivotal tool in isolating the specific allergens responsible for AP, enhancing the differential diagnosis process from other salivary gland disorders characterized by parotid swelling and discomfort.

## Abbreviations

AP, allergic parotitis: COP, chronic obstructive parotitis: CRP, chronic recurrent parotitis: SPT, skin puncture tests: FPT, food provocation test: ES, eosinophilic sialodochiti: OAS, oral allergy syndrome.

## Funding

The work reported in this paper was not supported by any fund.

## Availability of data and materials

The data that support the findings of this study are available on re-quest from the corresponding author. The data are not publicly avail-able due to privacy or ethical restrictions.

## Author contributions

S.G., S.L. and H.D. were involved in study design, acquisition of data, statistical analysis, interpretation of data, and drafted the manuscript; H.S., H.W., and Y.Y. were involved in statistical analysis and interpretation; X.D., J.Z., A.W., and X.C. were involved in acquisition and interpretation of data.

## Ethics approval and consent to participate

This study was approved by the Ethics Committee of Affiliated Stomatological Hospital of Nanjing Medical University. All the participants were informed about this study's objectives, risks, and benefits, and those who agreed to participate signed the free, informed consent form.

## Authors’ consent for publication

All authors agreed to the publication of this work in the World Allergy Organization Journal.

## Declaration of competing interest

The authors declare that they have no known competing financial interests or personal relationships that could have appeared to influence the work reported in this paper.
